# Understanding the Effects of Optimal Combination of Spectral Bands on Deep Learning Model Predictions: A Case Study Based on Permafrost Tundra Landform Mapping Using High Resolution Multispectral Satellite Imagery

**DOI:** 10.3390/jimaging6090097

**Published:** 2020-09-17

**Authors:** Md Abul Ehsan Bhuiyan, Chandi Witharana, Anna K. Liljedahl, Benjamin M. Jones, Ronald Daanen, Howard E. Epstein, Kelcy Kent, Claire G. Griffin, Amber Agnew

**Affiliations:** 1 Department of Natural Resources and the Environment, University of Connecticut, Storrs, CT 06269, USA; Chandi.witharana@uconn.edu (C.W.); amber.agnew@uconn.edu (A.A.); 2 Woodwell Climate Research Center, Falmouth, MA 02540, USA; aliljedahl@whrc.org; 3 Institute of Northern Engineering, University of Alaska Fairbanks, Fairbanks, AK 99775, USA; bmjones3@alaska.edu; 4 Alaska Department of Natural Resources, Division of Geological & Geophysical Surveys, Fairbanks, AK 99775, USA; ronald.daanen@alaska.gov; 5 Department of Environmental Sciences, University of Virginia, Charlottesville, VA 22904, USA; hee2b@virginia.edu (H.E.E.); kck7bw@virginia.edu (K.K.); cg4pm@virginia.edu (C.G.G.)

**Keywords:** deep learning, tundra, ice-wedge polygons, Mask R-CNN, satellite imagery, permafrost, Arctic

## Abstract

Deep learning (DL) convolutional neural networks (CNNs) have been rapidly adapted in very high spatial resolution (VHSR) satellite image analysis. DLCNN-based computer visions (CV) applications primarily aim for everyday object detection from standard red, green, blue (RGB) imagery, while earth science remote sensing applications focus on geo object detection and classification from multispectral (MS) imagery. MS imagery includes RGB and narrow spectral channels from near- and/or middle-infrared regions of reflectance spectra. The central objective of this exploratory study is to understand to what degree MS band statistics govern DLCNN model predictions. We scaffold our analysis on a case study that uses Arctic tundra permafrost landform features called ice-wedge polygons (IWPs) as candidate geo objects. We choose Mask RCNN as the DLCNN architecture to detect IWPs from eight-band Worldview-02 VHSR satellite imagery. A systematic experiment was designed to understand the impact on choosing the optimal three-band combination in model prediction. We tasked five cohorts of three-band combinations coupled with statistical measures to gauge the spectral variability of input MS bands. The candidate scenes produced high model detection accuracies for the F1 score, ranging between 0.89 to 0.95, for two different band combinations (coastal blue, blue, green (1,2,3) and green, yellow, red (3,4,5)). The mapping workflow discerned the IWPs by exhibiting low random and systematic error in the order of 0.17–0.19 and 0.20–0.21, respectively, for band combinations (1,2,3). Results suggest that the prediction accuracy of the Mask-RCNN model is significantly influenced by the input MS bands. Overall, our findings accentuate the importance of considering the image statistics of input MS bands and careful selection of optimal bands for DLCNN predictions when DLCNN architectures are restricted to three spectral channels.

## 1. Introduction

Automated image analysis has long been a challenging problem in multiple domains. Over the last decade, deep learning (DL) convolutional neural nets (CNNs) have reshaped the boundaries of computer visions applications (CV), enabling unparalleled opportunities for automated image analysis. Applications span from everyday image understanding through industrial inspections to medical image analysis [1]. Conspicuous shortfalls of traditional per-pixel based approaches when confronted with sub-meter scale remote sensing imagery (satellite and aerial) have shifted the momentum towards novels paradigms, such as object-based image analysis (OBIA) [2], in which homogenous assemblages of pixels are considered in classification process. Recently, OBIA has been flanked by the challenges of big data [3] and scalability [4]. The success of DLCNNs in CV applications has received great interest from the remote sensing community [5]. There has been an explosion of studies integrating DLCNN to address remote sensing classification problems spanning from general land use and land cover mapping [6,7] to targeted feature extraction [8,9,10]. Deep learning CNNs excel at object detection [10,11,12], semantic segmentation (multiple objects of the same class indicate a single object) [7,13], and semantic object instance segmentation (multiple objects of the same class indicates distinct individual objects) [14]. Over the years, a plethora of DLCNN architectures have been proposed, developed, and tested. The influx of new DLCNNs continues to grow. Each has its own merits and disadvantages with respect to the detection and/or classification problem at hand. The appreciation for DLCNNs in the remote sensing domain is increasing, while some of the facets that are unique to remote sensing image analysis have been overlooked along the way. 

Remote sensing scene understanding deviates from everyday image analysis in multiple ways, such as imaging sensors and their characteristics, coverage and viewpoints, and the objects and their behaviors in question. From the standpoint of Earth imaging, the image can be perceived as a reduced representation of the scene [2]. The image modality departs from the scene modality depending on the sensor characteristics. Scene objects are real-world objects, where image objects are assemblages of spatially arranged samples that model the scene. Images are only snapshots, and their size and shape are dependent on the sensor type and spatial sampling. For instance, certain land cover types, such as vegetation, are well-pronounced, exhibiting greater discriminative capacity in the near infrared (NIR) region than in the visible range. If the imaging sensor is constrained to the visible range, we limit ourselves from getting the advantage of the NIR wavelengths in classification algorithms. Similar to spectral strengths, the spatial resolution of the imaging sensor can either prohibit or permit our ability to construct the shape of the geo objects and their spatial patterning [15]. There is no single spatial scale that explains all the objects, but the semantics we pursue are organized into a continuum of scales [16,17]. In essence, an image represents the sensor’s view of the reality and not the explicit representation of scene objects.

These are practical challenges that apply to very high spatial resolution (VHSR) multispectral (MS) commercial satellite imagery. The luxury of VSHR satellite imagery is that the wavelengths are not confined to traditional panchromatic, standard red-green-blue (RGB) channels, or NIR. VSHR satellite imagery includes both visible and NIR regions and, therefore, produce an array of multiple spectral channels. For instance, the WorldView02 sensor captures eight MS channels at less than 2 m resolution, and data fusion techniques allow resolution-enhanced MS products at sub-meter spatial resolutions. Besides spatial details, discriminating one geo object from another could be straightforward or difficult depending on their spectral responses recorded in the MS channels. Selection of optimal spectral bands is a function of the type of environment and the kind of information pursued in the classification process. In remote sensing mapping applications, land cover types and their constituent geo objects exhibit unique reflectance behaviors in different wavelengths, or spectral channels, enabling opportunities to discriminate them from each other and characterize them into semantic classes. This leaves the question for the user to select the optimal spectral channels from the MS satellite imagery for DLCNN model applications. The decision is difficult when candidate DLCNN architectures restrain the input to only three spectral channels. An intriguing question is should one adhere to RGB channels while ruling out the criticality of other spectral bands, or is it necessary to mine all MS bands to choose the optimal bands for model predictions? To the best of our knowledge, this is a poorly explored problem despite its validity in remote sensing applications. Here, we make an exploratory attempt to understand this problem based on a case study that branches out from our on-going project on Arctic permafrost thaw mapping from commercial satellite imagery [18,19,20,21].

Permafrost thaw has been observed across the Arctic tundra [22]. Ice-rich permafrost landscapes commonly include ice wedges, for which growth and degradation is responsible for creating polygonized land surface features termed ice-wedge polygons (IWP). The lack of knowledge on fine-scale morphodynamics of polygonized landscapes introduces uncertainties to regional and pan-Arctic estimates of carbon, water, and energy fluxes [23]. Logistical challenges and high costs hamper field-based mapping of permafrost-related features over large spatial extents. In this regard, VHSR commercial satellite imagery enables transformational opportunities to observe, map, and document the micro-topographic transitions occurring in polygonal tundra at multiple spatial and temporal frequencies.

The entire Arctic has been imaged at 0.5 m resolution by commercial satellite sensors (DigitalGlobe, Inc., Westminster, CO, USA). However, imagery is still largely underutilized, and derived Arctic science products are rare. A considerable number of local-scale studies have analyzed ice wedge degradation processes using satellite imagery and manned-/unmanned aerial imagery/LiDAR data [24,25,26,27]. Most of the studies to date have relied on manual image interpretation and/or semi-automated approaches [25,26,28]. Therefore, there is a need and an opportunity for utilization of VHSR commercial imagery in regional scale mapping efforts to spatio-temporally document microtopographic changes due to thawing ice-rich permafrost. The bulk of remote sensing image analysis methods suffer from scalability and image complexities, but DLCNNs hold great promise in high throughput image analysis. Several pilot efforts [18,20,29,30,31] have demonstrated the potential adaptability of pre-trained DLCNN architectures in ice-wedge polygon mapping via the transfer learning strategy. However, the potential impacts of MS band statistics on DLCNN model predictions have been overlooked.

Owing to the increasing access of MS imagery and growing demand for a suite of pan-Arctic scale permafrost map products, there is a timely need to understand how spectral statistics of input imagery influence DLCNN model performances. Despite the design goals of DLCNN architectures to learn higher order abstractions of imagery without pivoting to the variations of low-level motifs, studies have documented the potential impacts of image quality, spectral/spatial artifacts of image compression, and other pre-processing factors on DLCNNs. Dodge et al. [32] described image quality impacts on multiple deep neural network models for image classification and showed that DL networks were sensitive to image quality distortions. Consequently, Dodge and Karam [33] carried out a comparison between human and deep learning recognition performance considering quality distortions and demonstrated that DL performance is still much lower than human performance on distorted images. Vasiljevic et al. [34] also investigated the effect of image quality on recognition by convolutional networks, which suffered a significant degradation in performance due to blurring along with a mismatch between training and input image statistics. Moreover, Karahan et al. [35] presented the influence of image degradations on the performance of deep CNN-based face recognition approaches, and their results indicated that blur, noise, and occlusion cause a significant decrease in performance. All these findings from previous studies provided useful insights towards developing computer visions applications, considering image quality that can perform reliably on image datasets.

Benchmark image datasets in CV applications are largely confined to RGB imagery, and trained DLCNNs are typically used for those data. Examples include imageNet, COCO, VisionData, MobileNet, etc. [36,37,38,39]. Priority for three spectral channels has become the de facto standard in everyday image analysis. As discussed before, this is a limiting factor in remote sensing applications. Training DLCNN architecture from scratch requires enormous amounts of training data to curtail overfitting. Because of this, transfer learning is becoming the standard practice to work with a limited amount of training data. In such circumstances, input channels are confined to three spectral channels, despite the original image containing more than three channels. In remote sensing, multispectral bands could significantly affect the capacity to be invariant to quality distortions. Selection of the optimal spectral band combination from all the available multispectral channels for model training and prediction is important because the dominant land cover types (heterogeneity) control the global image statistics as well as local spectral variance. Our contention is that improper band selection or reliance solely on RGB channels can potentially hamper mapping accuracies. The information content in MS channels should be prudently capitalized on DLCNN model predictions; otherwise; we are discarding valuable cues that are advantageous in automated detection and classification processes. The central objective of this exploratory study is to understand to what degree MS band statistics govern the DLCNN model predictions. We scaffold our analysis on a case study that includes ice-wedge polygons in two common tundra vegetation types (tussock and non-tussock sedge) as candidate geo objects. We choose Mask RCNN as the candidate DLCNN architecture to detect ice-wedge polygons from eight-band Worldview-02 commercial satellite imagery. A systematic experiment was designed to understand the impact of choosing the optimal three-band combination on model prediction. We tasked five cohorts of three-bands combinations coupled with statistical measures to gauge the spectral variability of input MS bands.

## 2. Study Area and Image Data

**Figure 1:**
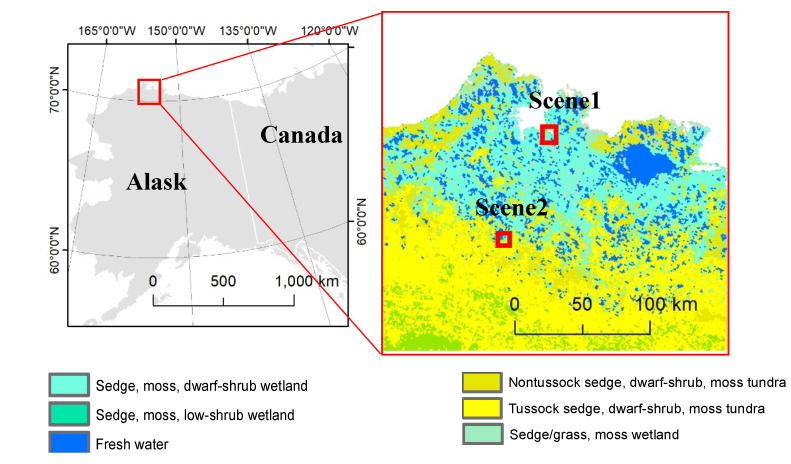
Location of candidate image scenes overlain on the Circumpolar Arctic Vegetation Map (CAVM, [40]).

Our study area covers coastal and upland tundra near Nuiqsut on the North Slope of Alaska (Figure 1). We obtained two summer-time WorldView-02 (WV2) commercial satellite image scenes from tussock sedge and non-tussock sedge tundra regions from the Polar Geospatial Center (PGC) at the University of Minnesota. The WV2 sensor records spectral reflectance at eight discrete wavelengths representing coastal blue (band 1), blue (band 2), green (band 3), yellow (band 4), red (band 5), red edge (band 6), NIR1 (band 7), and NIR2 (band 8). The spatial resolution of the data product is ~0.5 m with 16-bit radiometric resolution. Scenes were chosen from tussock and non-tussock sedge tundra regions based on the Circumpolar Arctic Vegetation Map (CAVM) [40], which presents important baseline reference data for pan-Arctic vegetation monitoring in tundra ecosystems [41]. Alaska has heterogeneous tundra types such as tussock sedge, dwarf shrub, and moss tundra in the foothills of northern Alaska [41]. The region generally covers (Figure 1, details in [40]): (i) Non-tussock sedge, dwarf-shrub, moss tundra: moist tundra dominated by sedges and dwarf shrubs < 40 cm tall, with a well-developed moss layer; (ii) Tussock sedge, dwarf-shrub, moss tundra: moist tundra, dominated by tussock cottongrass and dwarf shrubs <40 cm tall; (iii) Sedge: wetland complexes in the colder/warmer areas of the Arctic, dominated by sedges, grasses, and mosses. We chose two WV2 satellite image scenes focusing only two candidate vegetation types: (1) tussock sedge and (2) non-tussock sedge tundra for our analysis. 

## 3. Methodology

### 3.1. Mapping Workflow

**Figure 2:**
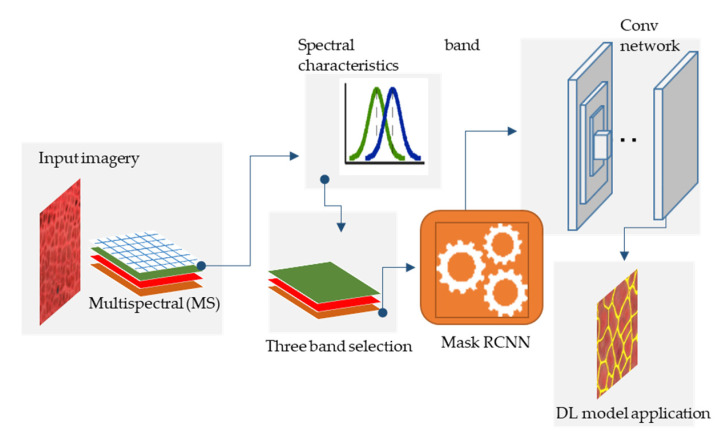
Simplified schematic of the automated ice wedge polygon mapping workflow.

In this study, we used a deep learning convolutional neural net (CNN) architecture called Mask-RCNN [42]. It is a semantic segmentation algorithm, which has been successfully applied in ice-wedge polygon mapping in Arctic regions [18,19,20,21]. The DL algorithm performs the object instance segmentation with outputs as predicted binary masks with classification information [18,19,20,21]. Our workflow has three stages. The first stage involves image pre-processing and tiling, in which the input image scene is partitioned into smaller subsets with dimensions of 200 pxl × 200 pxl. The second stage involves the application of the Mask RCNN model over the input tiles. In the last stage, the model predictions for each tile get post-processed and generate a seamless shapefiles corresponding to the input image scene. Figure 2 exhibits a generalized schematic diagram of the workflow.

We utilized ResNet-101 as the backbone of the Mask R-CNN model. The model was trained with a mini-batch size of two image tiles, 350 steps per epoch, learning rate of 0.001, learning momentum of 0.9, and weight decay of 0.0001. To minimize overfitting, random horizontal flips augmentation was applied to introduce variability in the training data for acceptable estimation accuracy. During calibration, the weights and biases of each neuron were estimated iteratively by minimizing a mean squared error cost function using a gradient descent algorithm with back propagation [43]. We exercised a transfer learning strategy. Pre-trained Mask RCNN was retrained using hand-annotated ice-wedge polygon samples generated from commercial satellite imagery. Approximately 40,000 ice-wedge polygon samples were incorporated. In the training schedule, the samples were divided into three categories of training (80%), validation (10%), and testing (10%) [18].

Like other commonly used DLCNNs, the pre-trained Mask RCNN is based on the training data repositories of everyday image information, such as coco data, which limits the use of more than three channels. In contrast, commercial satellite imagery captures substantial landscape heterogeneity based on an array of wavelengths, which may bias the model predictions. In this study, we chose two multispectral image scenes from varying tundra types: tussock and non-tussock sedge in our modeling approach. There is a unique opportunity to improve IWP mapping utilizing multispectral bands along with landscape information at regional scales. High spatial resolution is essential to accurately describe feature shapes and textural patterns, while high spectral resolution is needed to classify thematically detailed land-use and land-cover types [4,44]. Specifically, to ensure the spectral quality of the original image scene, the magnitude and variability of the band-wise measures in each pixel should be examined. This stage also helps to decrease the spectral heterogeneity, which identifies the best spectral band combination to produce a robust image classification model. In particular, to obtain the best combination of spectral bands from input multispectral imagery, we present three statistical measures: (1) probability distribution function (PDF), (2) cumulative distribution function (CDF), and (3) coefficient of variation (CV) [45,46,47,48,49,50,51,52,53]. To ensure the spectral quality of the original image scene, the magnitude and variability of the band-wise measure in each pixel were examined using these three statistical measures, which helps to decrease the spectral heterogeneity, leading to the best spectral band combination to produce a robust image detection model. 

As the first step in the pipeline, the most effective combination of bands is obtained by examining the shape of the cumulative density function (CDF) to control the number of pixel-based features for multispectral images and automatically determined a fixed number of features using CDF. Linag et al. [53] applied CDF along with a kernel density estimation method, which is used to obtain the detection threshold for the High-Resolution SAR Image application. Based on previous studies, we used CDF in our analysis to explain the distribution of the reflectance values among multiple spectral bands, and we choose the most similar three bands. Inamdar et al. [50] conducted a PDF matching technique for preprocessing of multitemporal remote sensing images for various applications, such as supervised classification, partially supervised classification, and change detection and successfully evaluated these techniques across scenarios. This study showed the necessity of matching distributions of two (or more) multispectral bands of remote sensing images for enhancing image modeling. Furthermore, Pitié et al. [51] also proposed a PDF-based algorithm to determine the possible change of content in image data to avoid excessive stretching of the mapping functions and successfully reduced the magnitude of the stretching. All these investigations motivate advancing the IWP mapping application by prudently mining multispectral bands using the PDF function. 

To examine the magnitude and variability of the band-wise measure, the coefficient of variation (CV—standard deviation divided by the mean) was examined to ensure the spectral quality of the original image scene [45,46,48]. To compare the degree of variation from one data series to another, we used the CV where we considered distributions with CV < 1 to be low variance, while we considered those with CV > 1 to be high variance. Previous studies used this metric to measure spectral variance to explore multispectral band influences in image processing applications. For example, Bovolo et al. [48] evaluated the effectiveness of their image processing approach in homogeneous and border areas using the CV in order to improve the geometric fidelity of mapping applications. Wang et al. [45] carried out an experiment using CVs for visible and near-infrared spectral regions across pixel sizes, and they found significant changes in the relative contribution to spectral diversity from leaf traits (detectable in the visible region) to canopy structure with increasing pixel size. To apply the CV in a preprocessing dataset, it helps to understand the spectral heterogeneity, which leads to a more robust image classification model than traditional object-detection models. Based on visual inspections, we confined ourselves to five cohorts of three-band combinations. Optimization of multispectral image bands based on CV, PDF, and CDF reduces the ambiguity, uncertainty, and errors in IWP mapping. The selected band combination had the maximum amount of information and the correlation between the bands was as small as possible. We also conducted a Kruskal-Wallis [54] test, where *p*-values among band combinations were calculated to see the significance of the accuracy assessment for each band combination. The Kruskal-Wallis test is a rank-based nonparametric test, which is used to determine significant differences among datasets. The Kruskal-Wallis test does not assume that the data are normally distributed, and this test allows us to compare more than two independent datasets [55]. The purpose of this statistical analysis is to systematically evaluate how the spectral character of the input data influences the prediction accuracies of DLCNN models.

### 3.2. Accuracy Estimates for Model Prediction

The DLCNN model assessment was performed using different error metrics: random error quantification (root mean square error, RMSE), systematic error quantification (absolute mean relative error, AMRE), and uncertainty quantification (F1 Score). The error metrics used in study are summarized below.

Absolute mean relative error (AMRE) is the mean of the relative percentage error, calculated by the normalized average:

(1) $$AMRE=\left|\frac{1}{n}\overset{n}{\underset{i=1}{\sum }}\left(\frac{{\hat{y}}_{i}-y_{i}}{y_{i}}\right)\right|$$

where the number of predicted polygons is $\;\hat{y}$, and the number of actual polygons is *y_i_*, and *n* is the quantity of samples used in the calculation. For an unbiased model, the AMRE would be 0 [56,57]. 

The root mean square error (RMSE) is a statistical metric used to measure the magnitude of the random error and defined as:

(2) $$RMSE=\sqrt{\frac{1}{n}\overset{n}{\underset{i=1}{\sum }}{\left[{\hat{y}}_{i}-y_{i}-\frac{1}{n}\overset{n}{\underset{i=1}{\sum }}\left({\hat{y}}_{i}-y_{i}\right)\right]}^{2}}$$

Here, RMSE ranges from 0 (an optimal value) to positive infinity [58,59].

Correctness indicates how many of the predicted positives were truly positive. Completeness determines what percentage of actual positives were detected. F1 Score derives a balance between Correctness and Completeness into one value [60]:

(3) $$\mathrm{Correctness}=\frac{\mathrm{TP}}{\mathrm{TP}+\mathrm{FP}}$$

(4) $$\mathrm{Completeness}=\frac{\mathrm{TP}}{\mathrm{TP}+\mathrm{FN}}$$

(5) $$F1\;\mathrm{Score}=\frac{2*\mathrm{Correctness}*\mathrm{Completeness}}{\mathrm{Completeness}+\mathrm{Completeness}}$$

Here, true positive (TP) is the number of polygons correctly identified, false positive (FP) is the number of polygons identified by model but not true, and false negative (FN) is undetected polygons. Higher values of Correctness indicate that there are fewer false positives in the classification. If the model always predicts positive magnitudes, Completeness will be 1, which indicates that ice-wedge polygons are properly detected by the model. Moreover, an F1 score of 1 indicates perfect prediction of ice-wedge polygons.

## 4. Results and Discussion

### 4.1. Statistical Measures for Input Image

**Figure 3:**
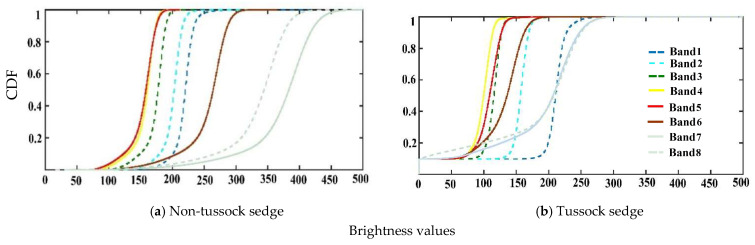
Cumulative distribution function for the multispectral band datasets.

The qualitative evaluation of the cumulative probability matching results is performed by visually comparing the shapes of the eight band distributions for tussock and non-tussock sedge tundra regions, which is shown in Figure 3. The CDF of spectral bands showed that band 7 and band 8 deviated significantly from the other bands for the non-tussock tundra sedge type (Figure 3). The band combination of 3,4,5 approximately presented similar spread and variability. 

**Figure 4:**
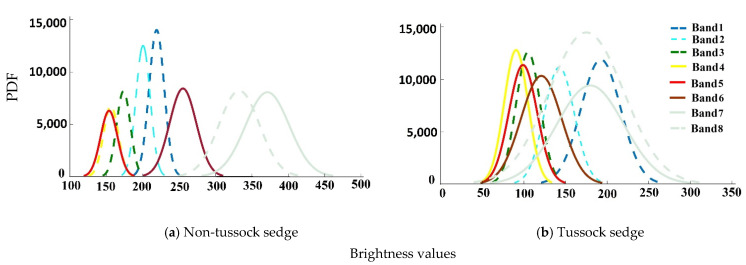
Probability distribution function for the multispectral band datasets.

Figure 4 shows the comparison of PDFs of the brightness values among all the spectral bands. Band 7 and band 8 displayed a clear separation of their PDFs from the other spectral bands for the non-tussock sedge tundra type. Specifically, it is interesting to note that bands 3,4,5 respond almost similarly compared to other spectral bands for both tundra types (tussock sedge and non-tussock sedge), highlighting the sensitivity of choosing band combination for the model predictions and its dependence on the brightness values. 

**Figure 5:**
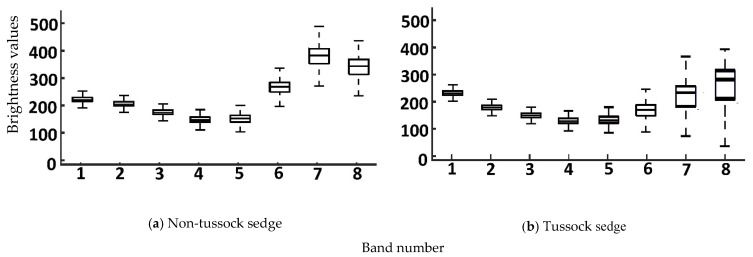
Box plot for the multispectral band datasets. In each box, the central mark is the median, and the edges are the first and third quartiles.

**Figure 6:**
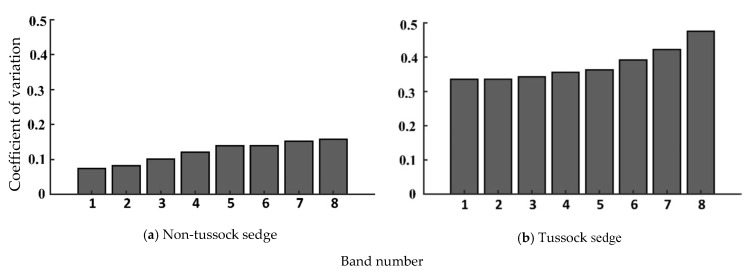
Coefficient of variation for the multispectral band datasets.

We produced box plots of the brightness values for spectral bands for tussock sedge and non-tussock sedge tundra types, as shown in Figure 5. There were no significant differences in terms of variability for the band combination of 1,2,3 and the band combination of 3,4,5, indicating that both combinations could be applied in model predictions. We used the coefficient of variation (CV) to evaluate the degree of variation of various spectral bands. From the analysis of CV from spectral bands, the distributions of brightness values showed low variability (CV < 1), which is shown in Figure 6. In terms of CV, the band combination of 1,2,3 and the band combination of 3,4,5 were consistent for tussock and non-tussock sedge tundra regions. These results helped us to understand the feasibility and reliability of the remote sensing information extraction for large-scale application research.

### 4.2. Statistical Measures for Model Prediction

We statistically evaluated the performances of the DL model in detecting IWPs. For the quantitative assessments, from each image scene, we randomly selected 15 subsets to manually delineate polygons as a reference (ground-truth polygons). Systematic error was exhibited for all image scenes, and results showed significantly low AMRE values (0.16–0.19) for three-band combinations (1,2,3 and 3,4,5) for the different tundra types (tussock and non-tussock sedge) (Table 1). Other combinations produced comparatively high systematic error (0.33–0.38). The results provide insights on how to select a proper band combination for remote sensing satellite images for deep learning modeling. Similarly, the random error for two band combinations (1,2,3 and 3,4,5) varied from 0.17 to 0.21 for the two candidate scenes (Table 2), indicating a robust performances of the new version of the ice-wedge polygon mapping algorithm in varying tundra types (tussock and non-tussock sedge). Therefore, this methodology adopted the best combination scheme to perform the image analysis for IWP mapping.

**Figure 7:**
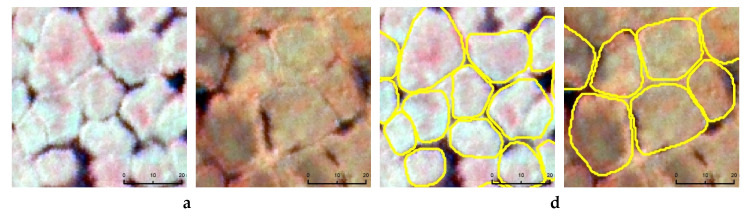

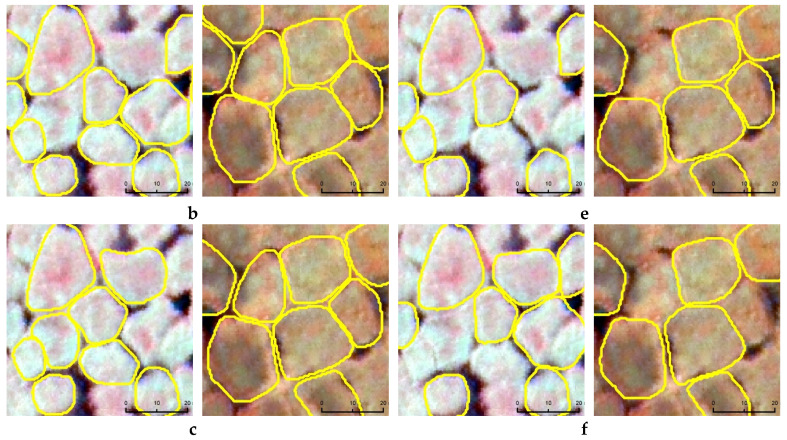
Simplified zoomed-in views of original imagery and model predictions. (**a**) original imagery for non-tussock sedge (form left column 1) and original imagery for tussock sedge (from left column 2); band combination (**b**) (1,2,3); (**c**) (2,3,5); (**d**) (2,3,7); (**e**) (3,4,5); (**f**) (3,5,7). Yellow outlines denote automatically detected IWPs. Imagery © [2012,2015] DigitalGlobe, Inc. (Westminster, CO, USA).

We used three quantitative error statistics (correctness, completeness, and F1 score) to show the performances of the framework. The candidate scenes 1 and 2 produced high model detection accuracies for the F1 score, ranging between 0.89 to 0.95, for two band combinations (1,2,3 and 3,4,5) (Figure 7, Table 3). Similarly, scenes 1 and 2 scored high values for completeness (85–91%). In the two cases, the correctness metric scored ~1, allowing less freedom for false alarms. Optimization of the multispectral bands improved the detection accuracy of the model.

**Figure 8:**
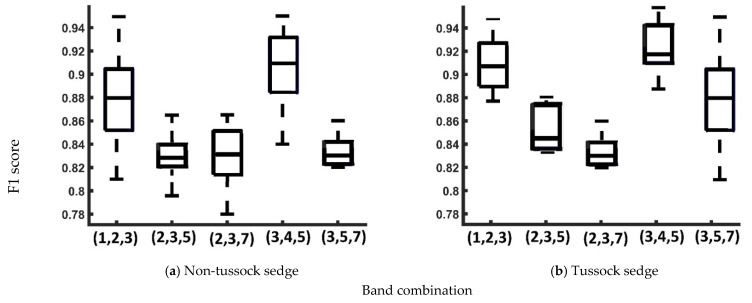
Box plot of F1 score for the different band combinations. In each box, the central mark is the median, and the edges are the first and third quartiles.

Box plots for the F1 score for five three-band cohorts (1,2,3; 2,3,5; 2,3,7; 3,4,5; and 3,5,7) are presented in Figure 8. The distribution of F1 scores showed substantial discrepancies in terms of variability for the band combinations for (2,3,5), (2,3,7), and (3,5,7) compared to other band combinations ((1,2,3) and (3,4,5)). Therefore, the two band combinations (1,2,3) and (3,4,5) are treated as the best combinations of spectral bands to create robust models for ice-wedge polygon mapping.

The Kruskal–Wallis test was conducted to determine whether the medians from various datasets are different or not. We calculated *p*-values for two band combinations (1,2,3 and 3,4,5) compared with other combinations to test for significant differences in F1 scores (Table 4). The *p*-values were found to be < α (alpha) = 0.05 for the combinations of (2,3,5), (2,3,7), and (3,5,7). On the other hand, the two band combinations (1,2,3) and (3,4,5) were not statistically different with *p*-values > α = 0.05. Therefore, the two band combinations (1,2,3) and (3,4,5) are used in deep learning-based modeling for this study area.

In this exploratory study, we use two tundra vegetation types, tussock sedge and non-tussock sedge. However, arctic tundra includes additional vegetation types. Therefore, the model can be biased when it is applied to other tundra vegetation types such as prostrate dwarf-shrub, herb, lichen tundra; rush/grass, forb, cryptogam tundra; graminoid, prostrate dwarf-shrub, forb tundra, etc. In the future, this experiment can be extended considering more diverse tundra landscapes, such as graminoid and shrub dominated vegetation cover types, to systemically gauge the improvement of the DL model prediction accuracies. 

The mapping workflow discerned the IWPs by exhibiting significantly low random and systematic error for band combinations (1,2,3). Results suggest that the prediction accuracy of the Mask-RCNN model is significantly influenced by the input MS bands. The DL model exhibited high detection accuracies (89% to 95%) compared to previous studies [20,21]. According to the results, it is also suggested that the removal of the unnecessary bands information is crucial for enhancing the model accuracy. Most of the DLCNN architectures are tailored to operate in the context of three-band or single-band images. Therefore, when practicing transfer-learning strategies, there is a high chance of overlooking the wealth of information contained in multispectral image channels and their correlation among bands. This additional information could prudently leverage model performances and prediction accuracy. Overall, transfer-learning strategies optimized the model with limited data utilizing processing time and computation resources instead of original DL based hardcore programming competencies. Moreover, the use of all spectral bands does not necessarily improve classification accuracy, but there is a high tendency to degrade to prediction accuracies. The sensible approach is to methodologically select optimal three bands cohort with respect to the classification problem in hand. Therefore, to create an automated ice-wedge polygon mapping framework, optimal combination of spectral bands was obtained from the very high resolution satellite imagery. In summary, we conducted our exploratory analysis based on transfer learning strategy to get the most benefit out from existing CNN weights that are formulated based on three-band imagery from everyday image analysis. While the current exploratory study scaffolds on a single candidate DL model, a candidate geo-object, and a satellite sensor model, in future research, we will aim to expand the experimental design incorporating multiple DL models, a suite of geo-object types, and several satellite sensor model to investigate the effect of band statistics on DL model predictions. 

## 5. Conclusions

Our study highlights the importance of the band combinations in the use of multispectral datasets on deep learning convolutional neural net (DLCNN) model prediction accuracy. Here, in the context of ice-wedge polygon mapping, we applied the DLCNN architecture, to detect IWPs from eight-band Worldview-02 VHSR satellite imagery in the North slope of Alaska across two common tundra vegetation types (tussock sedge and non-tussock sedge tundra). We found that the prediction accuracy of the Mask-RCNN model is significantly influenced by the input of MS bands. Optimization of multispectral image bands combination reduced the ambiguity, uncertainty, and errors in ice-wedge polygon mapping. Results suggest that mapping applications depend on the careful selection of the best band combinations. The selected band combination had the maximum amount of information with the least amount of correlation among the bands and the correlation between the bands were as small as possible. Overall, our findings emphasize the importance of considering the image statistics of input MS bands and careful selection of optimal bands for DLCNN predictions, when the architectures are restricted with three spectral channels. Thinking beyond yet another target detection based on transfer learning, we have materialized an-image-to-assessment pipeline, which is capable of parallel processing of sheer volumes of image scenes on HPC resources. Our framework is not fundamentally limited to IWP mapping but rather an extensible workflow to perform high throughput, regional-scale mapping tasks such as other permafrost features; thaw slumps, capillaries, shrubs, etc. Our future study is to apply the mapping algorithm over large land areas of the heterogeneous Arctic tundra.

## Figures and Tables

**Table 1:** Summary statistics of Absolute Mean relative Error (AMRE) for candidate scenes.

Image Scene	Three Band Combination				
	1,2,3	2,3,5	2,3,7	3,4,5	3,5,7
Non-tussock sedge	0.17	0.35	0.33	0.16	0.38
Tussock sedge	0.19	0.38	0.35	0.19	0.38

**Table 2:** Summary statistics of Root Mean Square Error (RMSE) for candidate scenes.

Image Scene	Three Band Combination				
	1,2,3	2,3,5	2,3,7	3,4,5	3,5,7
Non-tussock sedge	0.20	0.38	0.34	0.19	0.42
Tussock sedge	0.21	0.39	0.35	0.17	0.47

**Table 3:** Accuracy assessment of detection for candidate image scenes.

Band Combination	Non-Tussock Sedge			Tussock Sedge		
	Correctness	Completeness	F1 Score	Correctness	Completeness	F1 Score
1,2,3	1	85%	0.89	1	89%	0.92
2,3,5	1	81%	0.84	1	82%	0.85
2,3,7	1	82%	0.84	1	82%	0.83
3,4,5	1	86%	0.91	1	91%	0.95
3,5,7	1	82%	0.83	1	82%	0.88

**Table 4:** *p*-values of the Kruskal–Wallis test for F1 scores.

Non-Tussock Sedge			Tussock Sedge		
Band Combination	1,2,3	3,4,5	Band Combination	1,2,3	3,4,5
1,2,3	1	0.0824	1,2,3	1	0.0189
2,3,5	4.22 × 10^−4^	2.33 × 10^−5^	2,3,5	0.0	0.0
2,3,7	0.0011	4.01 × 10^−5^	2,3,7	0.0	0.0
3,4,5	0.0824	1	3,4,5	0.0189	1
3,5,7	3.77 × 10^−4^	7.83 × 10^−5^	3,5,7	1.40 × 10^−3^	4.11 × 10^−5^
